# Incidental Detection of Neoplastic B Cells in the Colon Following Resection in a Patient With Adenocarcinoma

**DOI:** 10.14309/crj.0000000000001844

**Published:** 2025-09-29

**Authors:** Asrita Vattikonda, Hasan Saleh, Ian Dryden, Maoyin Pang

**Affiliations:** 1 Department of Internal Medicine, Mayo Clinic, Jacksonville, FL; 2 Department of Laboratory Medicine and Pathology, Mayo Clinic, Jacksonville, FL; 3 Division of Gastroenterology and Hepatology, Mayo Clinic, Jacksonville, FL

**Keywords:** monoclonal B-cell lymphocytosis, colonoscopy, adenocarcinoma, chronic lymphocytic leukemia

## Abstract

Following a routine screening colonoscopy, a 54-year-old man underwent colon resection for adenocarcinoma, which revealed neoplastic B-cells in the lymph nodes surrounding the colon suggestive of chronic lymphocytic leukemia (CLL). Further evaluation identified a monoclonal B-cell population, and he was diagnosed with monoclonal B-cell lymphocytosis without meeting CLL diagnostic criteria. The patient had no clinical signs of leukemia and did not need acute treatment but was placed under annual surveillance. This case highlights the incidental discovery of neoplastic B-cells, emphasizing the importance of comprehensive histopathological evaluation and clinical correlation to guide management and monitoring for potential progression to CLL.

## INTRODUCTION

Neoplastic B cells are rarely found in the colon without a previous diagnosis of chronic lymphocytic leukemia (CLL) or monoclonal B-cell lymphocytosis (MBL). Gastrointestinal involvement in CLL is typically associated with advanced disease.^1,2^ Primary colonic lymphomas, such as mucosa-associated lymphoid tissue lymphomas and diffuse large B-cell lymphomas are rare, although they can sometimes provide context for the presence of neoplastic B-cells in the colon.^3,4^We report the case of a 54-year-old man with no medical history who underwent colon resection for adenocarcinoma, with hematopathologic findings concerning for CLL based on the presence of an incidental neoplastic B-cell population.

## CASE REPORT

**Figure 1.**
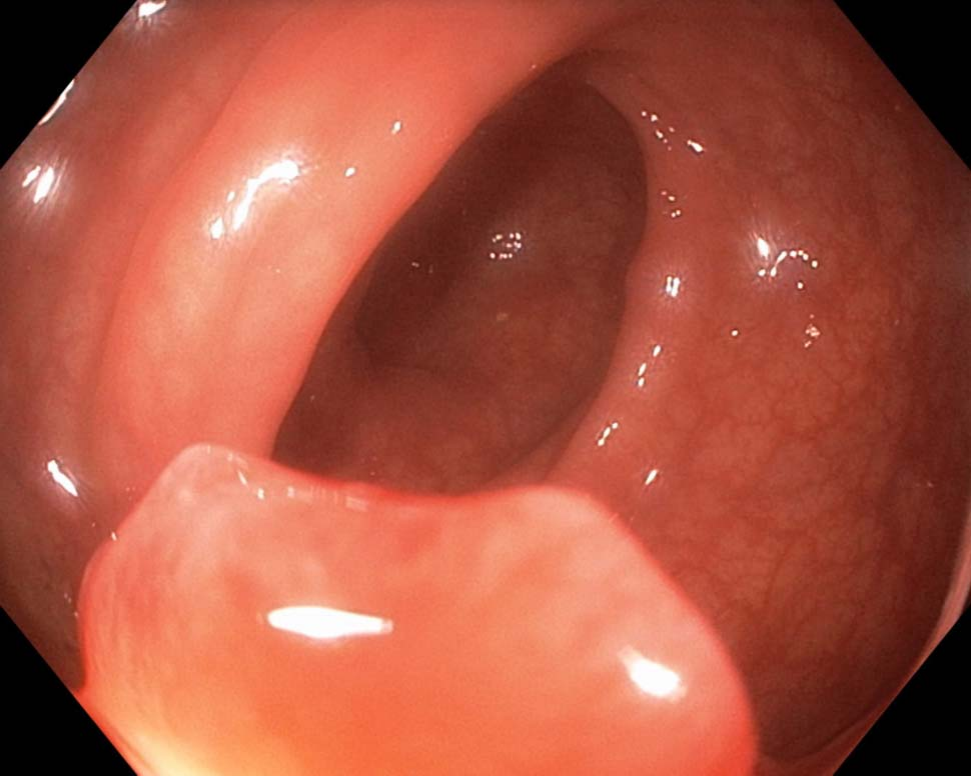
Endoscopic image of a 1-cm pedunculated polyp in the rectosigmoid colon, located 19 cm from the anal verge. The polyp demonstrated central surface depression concerning for malignancy. It was resected completely with a hot snare, followed by placement of a hemostatic clip and submucosal tattooing for localization. Final pathology revealed poorly differentiated adenocarcinoma.

A 54-year-old male patient with no significant past medical history presented initially for a general medical examination. He reported feeling well overall apart from some mild upper abdominal and groin discomfort. Physical examination, vital signs, and routine laboratory tests were all normal. He did not have a family history of colon cancer. He soon underwent his first screening colonoscopy, where 4 polyps were found in the rectosigmoid colon and subsequently resected. One was a 10-mm polyp that was pedunculated with central depression, and a tattoo was placed for localization due to concern for malignancy (Figure 1). Pathology from the high-risk polyp showed adenocarcinoma, poorly differentiated, arising in a background of tubular adenoma with high-grade dysplasia. There were multiple fragments, so the depth of invasion was unable to be assessed. Mismatch repair immunochemistry demonstrated normal expression of *MLH1*, *MSH2*, *MSH6*, and *PMS2*. The patient underwent cross-sectional imaging, which revealed lymphadenopathy in the inguinal, hilar, and axillary regions favored to be inflammatory or reactive.

**Figure 2.**
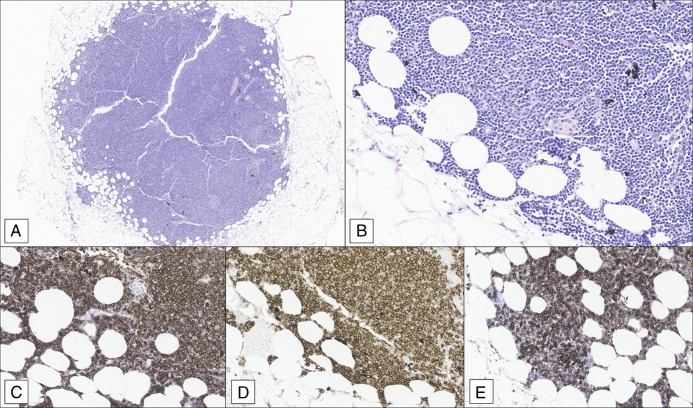
(A) One lymph node with rare diminutive germinal centers, partially effaced nodal architecture, and indistinct capsular border. Black pigmented macrophages containing tattoo ink noted in the background (H&E, 20×). (B) Enlarged neoplastic lymphocytes with atypical nuclei infiltrate surrounding adipose tissue located in the lymph nodes in the surrounding soft tissue (H&E, 200×). (C–E) Neoplastic B-cell lymphocytes in the lymph nodes in the mesenteric soft tissue surrounding the colon are positive for CD20, CD23, and CD5 by immunohistochemistry (IHC, 200×).

The patient underwent colorectal surgery evaluation for colon cancer with no lymph node involvement or metastasis. He underwent a flexible sigmoidoscopy, which confirmed the location of the tattoo at 20 cm in the sigmoid colon. Shortly thereafter, he underwent robotic-assisted anterior sigmoid colonic resection. Surgical pathology was negative for residual adenocarcinoma in the colonic tissue but revealed findings in the surrounding lymph nodes concerning for CLL/small lymphocytic lymphoma. The lymph nodes had ill-defined capsular borders with a loss of normal nodal architecture, suggestive of a clonal lymphoproliferative process. Immunohistochemistry performed on the lymph nodes demonstrated a neoplastic B-cell population positive for CD20, CD5, and CD23, and negative for CD3, CD10, and Cyclin D1 (Figure 2).

The patient was swiftly referred to hematology for comprehensive evaluation. Peripheral blood flow cytometry was performed and showed a kappa light chain restricted B-cell population with immunophenotypic features of CLL/small lymphocytic lymphoma, but the size of the population (1.32 × 10^9^/L cells) did not meet quantitative criteria for CLL as it was under 5,000 clonal B-cells/µL. Fluorescence in situ hybridization evaluating for CLL was normal and next-generation sequencing was negative for p53 mutation. He did not have any cytopenias, constitutional symptoms, hepatosplenomegaly, or lymphadenopathy (>1.5 cm), and therefore there was no need for treatment of a possible CLL. He was then diagnosed with low-count MBL, a premalignant condition, given the evidence of neoplastic B cells in the colon. He was counseled on signs of progression to CLL. The patient continued to progress well postoperatively at colorectal surgery and hematology follow-up visits. He was placed under annual surveillance with clinical examinations and laboratory testing.

## DISCUSSION

This is a rare case of a patient with a neoplastic B-cell population in the colon, incidentally discovered during sigmoid colon resection of primary sigmoid colon adenocarcinoma. Though the hematopathologic evaluation was highly concerning for CLL, clinical correlation was recommended and the patient was diagnosed with low-count MBL following comprehensive evaluation. MBL is characterized by the presence of a monoclonal B-cell population with immunophenotype of CLL that does not meet the diagnostic criteria for CLL, as defined by the National Comprehensive Cancer Network.^5^ The frequency of MBL increases with age and is 3.5%–6.7% in those aged 40–60 years. MBL can be categorized as either low-count or high-count based on a B cell count below or above 0.5 × 10^9^/L. Low-count MBL rarely progresses to CLL, while high-count MBL progresses to CLL requiring treatment at a rate of 1%–2% annually. Signs of progression to CLL include doubling of white blood cell count, anemia, thrombocytopenia, >1 month of night sweats, significant fatigue or weight loss, enlarged lymph nodes or spleen, and recurrent infections. Additional prognostic markers such as CD38 expression and cytogenetic abnormalities may further inform risk stratification in high-count MBL, although their utility in low-count MBL remains limited. If a patient does not show any clinical evidence of progression to CLL, annual surveillance with a complete blood count and lymph node examination is advised. There is no additional need for screening colonoscopy outside of regular surveillance.^6^

MBL is not uncommon in the general population and can be detected in 5% to >20% of adults over the age of 60, though most cases are diagnosed through peripheral blood testing during evaluation of lymphocytosis or lymphadenopathy. MBL is especially unique in gastrointestinal specimens without systemic disease.^6–8^ Colon involvement typically occurs in patients with advanced or progressive CLL, and neoplastic B cells are rarely found in primary colonic lymphoma, such as mucosa-associated lymphoid tissue lymphoma or diffuse large B-cell lymphomas.^1–4,9^ Currently, there are no previous reports of MBL detected incidentally through histologic evaluation of a resected colon adenocarcinoma specimen.

This case highlights an unusual histopathologic finding of neoplastic B cells identified following resection of a screening-detected colon adenocarcinoma. The patient received timely and appropriate care, including early specialist referrals that clarified the diagnosis and excluded other concerning conditions, leading to a favorable outcome. The discovery of neoplastic B cells in the colon prompted further evaluation and the finding of a monoclonal B-cell population in the peripheral blood, ultimately leading to the diagnosis of MBL. Recognizing such findings enables appropriate long-term monitoring and fosters clinical vigilance for potential progression to CLL. This case underscores the importance of thorough histopathological evaluation of incidental findings and the need for comprehensive clinical correlation to guide patient management.

## DISCLOSURES

Author contributions: A. Vattikonda managed the patient data, conducted literature review, and drafted the manuscript. H. Saleh conducted literature review and critically reviewed the manuscript. I. Dryden interpreted pathology findings, contributed to figure preparation, and critically reviewed the manuscript. M. Pang identified the case and critically reviewed the manuscript. A. Vattikonda is the article guarantor.

Financial disclosure: None to report.

Informed consent was obtained for this case report.
